# The emerging GII.P16-GII.4 Sydney 2012 norovirus lineage is circulating worldwide, arose by late-2014 and contains polymerase changes that may increase virus transmission

**DOI:** 10.1371/journal.pone.0179572

**Published:** 2017-06-29

**Authors:** Christopher Ruis, Sunando Roy, Julianne R. Brown, David J. Allen, Richard A. Goldstein, Judith Breuer

**Affiliations:** 1 Division of Infection and Immunity, University College London, London, United Kingdom; 2 Department of Microbiology, Virology and Infection Control, Great Ormond Street Hospital for Children, London, United Kingdom; 3 Department of Pathogen Molecular Biology, Faculty of Infectious and Tropical Diseases, London School of Hygiene & Tropical Medicine, London, United Kingdom; 4 Virus Reference Department, National Infections Service, Public Health England, London, United Kingdom; 5 NIHR Health Protection Research Unit in Gastrointestinal Infections, United Kingdom; National Institute of Allergy and Infectious Diseases, UNITED STATES

## Abstract

Noroviruses are a leading cause of human gastroenteritis worldwide. The norovirus genotype GII.4 is the most prevalent genotype in the human population and has caused six pandemics since 1995. A novel norovirus lineage containing the GII.P16 polymerase and pandemic GII.4 Sydney 2012 capsid was recently detected in Asia and Germany. We demonstrate that this lineage is also circulating within the UK and USA and has been circulating since October 2014 or earlier. While the lineage does not contain unique substitutions in the capsid, it does contain polymerase substitutions close to positions known to influence polymerase function and virus transmission. These polymerase substitutions are shared with a GII.P16-GII.2 virus that dominated outbreaks in Germany in Winter 2016. We suggest that the substitutions in the polymerase may have resulted in a more transmissible virus and the combination of this polymerase and the pandemic GII.4 capsid may result in a highly transmissible virus. Further surveillance efforts will be required to determine whether the GII.P16-GII.4 Sydney 2012 lineage increases in frequency over the coming months.

## Introduction

Noroviruses are the leading cause of human gastroenteritis worldwide and are estimated to be responsible for 900,000 clinic visits amongst children in industrialized countries and up to 200,000 deaths of children in developing countries annually [1,2]. Noroviruses belong to the *Caliciviridae* family and their ~7.5Kb RNA genome contains three open reading frames (ORFs): ORF1 encodes a nonstructural polyprotein that is cleaved into six proteins including an RNA-dependent RNA polymerase (RdRp), ORF2 encodes the VP1 capsid protein and ORF3 encodes a minor structural protein, VP2. Recombination frequently occurs close to the junction between ORF1 and ORF2, necessitating independent genotyping of the RdRp and capsid [3]. While noroviruses are divided into seven genogroups and further into more than 30 genotypes based on capsid sequence, the majority of cases and outbreaks are caused by viruses associated with a single capsid genotype, GII.4, which has also caused six pandemics of gastroenteritis since 1995 [4,5]. Each pandemic has been caused by a distinct strain of GII.4. While the first five pandemic strains contained the GII.P4 RdRp, the most recent pandemic strain (Sydney 2012) circulated more commonly with the GII.Pe RdRp. Recent reports demonstrated circulation of the Sydney 2012 capsid with a GII.P16 RdRp in South Korea, Japan and Germany [6–8]. While the GII.P16 RdRp is not typically highly prevalent, a GII.P16-GII.2 virus was the dominant strain amongst a large peak of norovirus infections in Germany in Winter 2016 [8]. Here, we demonstrate using whole genome sequencing [9] and phylogenetic analyses that the GII.P16-GII.4 Sydney 2012 lineage is also circulating within the UK and USA. While the lineage does not contain unique capsid substitutions, it does contain RdRp substitutions that are shared with the GII.P16-GII.2 RdRp and are close to positions known to influence RdRp function and viral transmission.

## Materials and methods

### Sample collection and sequencing

We identified noroviruses with the GII.P16 RdRp in ten stool samples collected as part of routine surveillance from South East and North West England between June 2015 and April 2016; samples were from both sporadic cases and outbreaks. Four of these faecal specimens were referred to the Virus Reference Department, Public Health England, as part of a sentinel norovirus strain surveillance programme, which collects norovirus-positive specimens from geographically disparate regions across England. The other six faecal specimens were collected from a tertiary referral paediatric hospital in London, UK. These six specimens were residual diagnostic specimens obtained from patients with confirmed norovirus infections. Specimens were collected as part of the FP7 PATHSEEK study and submitted to the UCL Infection DNA Bank. The samples were supplied to the study in an anonymised form; the use of these specimens for research was approved by the NRES Committee London—Fulham (REC reference: 12/LO/1089). Other specimens used were sent to the Enteric Virus Unit at Public Health England in the course of routine surveillance and diagnosis work. RNA was extracted and whole genome sequencing performed as described previously [9]. Sample genotypes were obtained using the norovirus genotyping tool, available at http://www.rivm.nl/mpf/norovirus/typingtool [10]. The GenBank accession numbers for viruses sequenced in this study are as follows: KY887597-KY887606.

### Phylogenetic analyses

We combined our sequences with all GII.P16 ORF1 sequences and all GII.4 Sydney 2012 capsid and VP2 sequences available on GenBank. We reconstructed maximum likelihood trees using RAxML [11] and time trees using BEAST 2 [12]. GII.P16 dating analyses were carried out using the RdRp as there are many more GII.P16 sequences containing the RdRp (n = 165) compared with the complete ORF1 (n = 45), enabling estimation of more accurate dates. The GII.4 Sydney 2012 capsid maximum likelihood tree was used to identify a well-supported monophyletic clade (bootstrap support 81) containing 70 samples that includes all of the samples with the GII.P16 RdRp. The GII.4 Sydney 2012 time tree was reconstructed using the samples in this clade. Ancestral reconstruction to identify nonsynonymous changes occurring along particular branches was carried out using PAML [13].

### Data availability

All alignments, phylogenetic trees and BEAST XML files are included as supporting information (S1–S6 Files).

## Results

**Fig 1:**
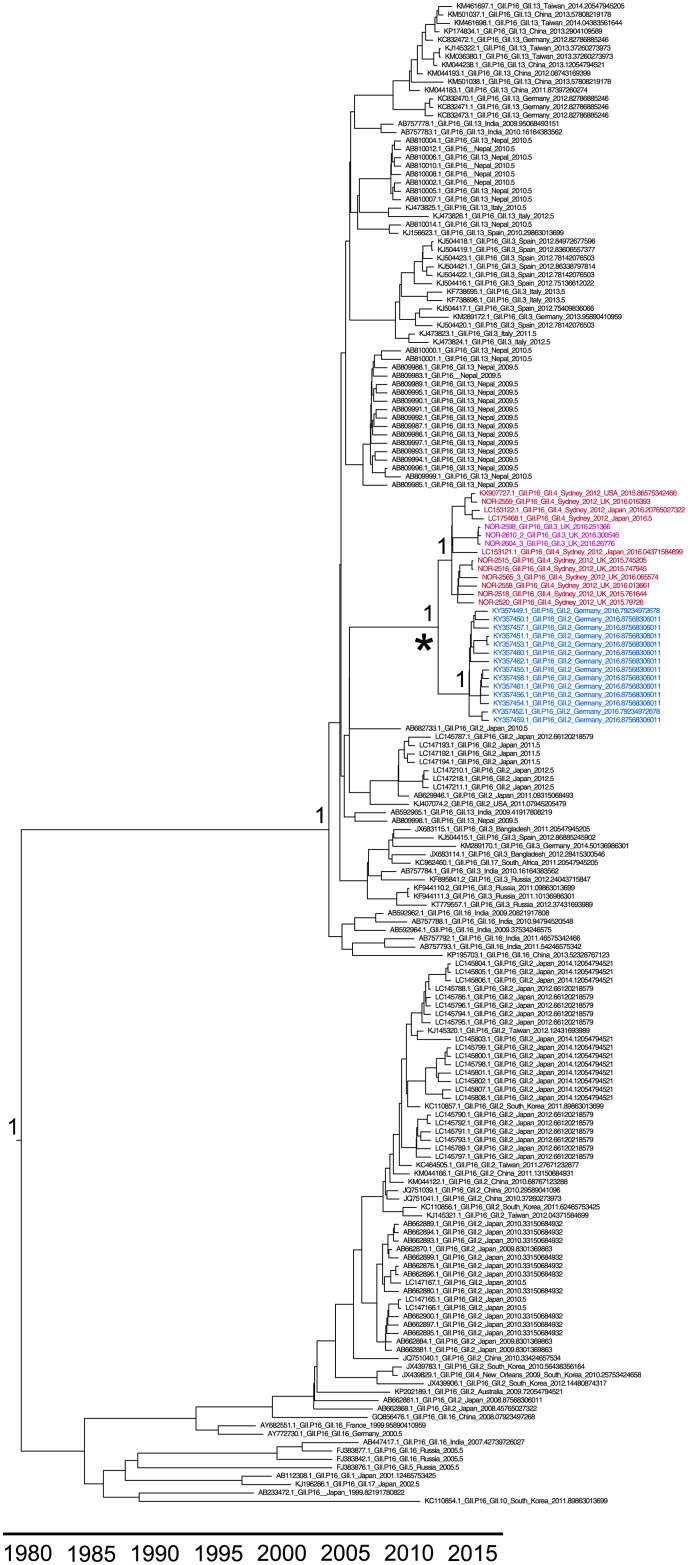
Evolutionary history of the GII.P16 lineage. Time tree of 179 GII.P16 RdRp sequences reconstructed using BEAST 2. The GII.P16 samples found with the GII.4 Sydney 2012 capsid are highlighted red. The GII.P16 samples found with the GII.3 capsid are highlighted in magenta. The GII.P16-GII.2 samples associated with most norovirus cases in Germany in winter 2016 are highlighted in blue. The starred node is the common ancestor of this clade. Posterior supports are shown at key nodes.

We identified ten viruses collected in routine surveillance in the UK containing the GII.P16 RdRp. Of these, seven were found with the GII.4 Sydney 2012 capsid and three were found with the GII.3 capsid. The ten RdRp sequences formed a well-supported monophyletic clade (Fig 1), that also contains GII.P16-GII.4 Sydney 2012 samples from the USA and Japan, including the GII.P16-GII.4 Sydney 2012 sample described in 2016 from Kawasaki City, Japan [7]. These samples also cluster in the phylogenetic tree of available GII.P16 ORF1 samples. The GII.P16-GII.4 Sydney 2012 RdRps cluster with the GII.P16-GII.2 RdRps that dominated norovirus outbreaks in Germany in winter 2016 (Fig 1) [8]. The common ancestor of the GII.P16-GII.4 Sydney 2012 and GII.P16-GII.2 RdRps occurred in March 2013 (95% highest probability density (HPD) January 2012-May 2014).

**Fig 2:**
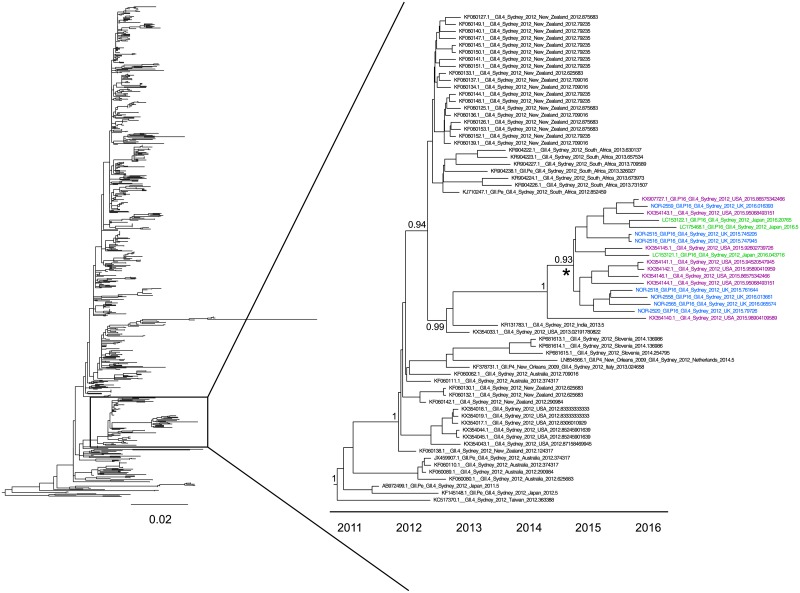
Evolutionary history of the GII.4 Sydney 2012 capsid. A maximum likelihood tree was reconstructed for 781 GII.4 Sydney 2012 capsid samples. From this, a well supported clade (bootstrap support 81) containing the GII.P16-GII.4 Sydney 2012 lineage and several other samples was selected and a time tree reconstructed on this smaller dataset using BEAST 2. The starred node is the common ancestor of the GII.P16-GII.4 Sydney 2012 lineage and the samples in this lineage are coloured based on the country from which they were isolated: blue—UK, purple—USA, green—Japan. Posterior supports are shown at key nodes.

In a phylogenetic tree containing all available GII.4 Sydney 2012 capsid sequences, the GII.P16-GII.4 Sydney 2012 samples from the UK again cluster with samples from the USA and Japan containing the GII.P16 RdRp (Fig 2). This clade contains additional samples from the USA where the RdRp was not sequenced. However, all of the RdRps sequenced within this clade are of the GII.P16 genotype. It is therefore likely that these samples also contain the GII.P16 RdRp, although without genotyping this is uncertain. The common ancestor of this clade occurred in October 2014 (95% HPD June 2014-February 2015).

**Fig 3:**
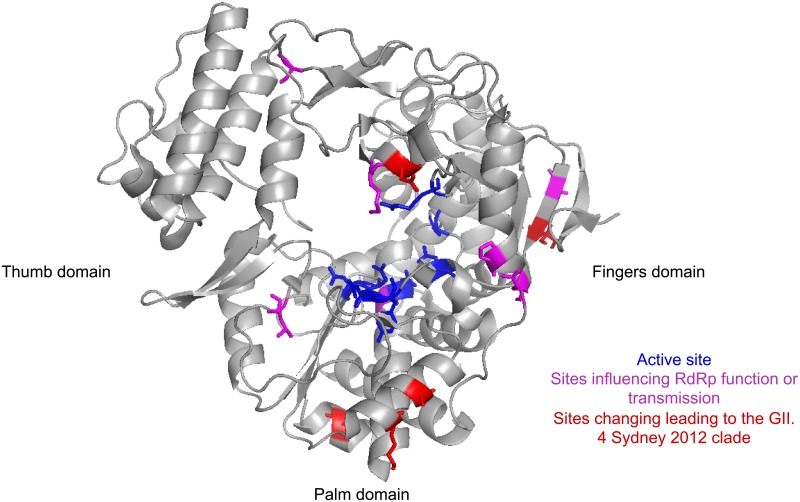
Location of RdRp sites that change leading to the GII.P16-GII.4 Sydney 2012 clade. Sites are highlighted on PDB structure 1SH0. The sites that change leading to the GII.P16-GII.4 Sydney 2012 clade are shown in red. The sites that form the RdRp active site are shown in blue. Sites previously demonstrated to alter RdRp function and/or transmission when mutated are shown in magenta (14,15).

No nonsynonymous substitutions occurred in the capsid along the branch leading to the GII.P16-GII.4 Sydney 2012 clade and there are no amino acid changes shared amongst capsids in this clade that are not found in the remainder of the GII.4 Sydney 2012 capsids. However, 14 nonsynonymous substitutions occurred within ORF1 along the branch leading to the common ancestor of the GII.P16-GII.4 Sydney 2012/GII.3 clade (Fig 1, Table 1). Eleven of these changes are shared amongst all of the samples in the GII.P16-GII.4 Sydney 2012/GII.3 clade. Five of these changes occurred in the RdRp, with several of the changes occurring close to positions known to impact polymerase function and transmission (Fig 3) [14,15]. Only four of the positions that change leading to the GII.P16-GII.4 Sydney 2012/GII.3 clade are in the partial RdRp region sequenced by Niendorf et al in the GII.P16-GII.2 samples [8]. All four of these nonsynonymous changes are also present in the GII.P16-GII.2 samples and were therefore acquired by the common ancestor of the RdRp clade containing the samples with the GII.4 Sydney 2012, GII.3 and GII.2 capsids. Additionally, a single nonsynonymous change (S157N) occurred in VP2 along the branch leading to the GII.P16-GII.4 Sydney 2012 clade.

**Table 1:** Sites in ORF1 that change leading to the GII.P16-GII.4 Sydney 2012 clade.

Change	Protein	Polymerase position	Shared by all GII.P16-GII.4 Sydney 2012 samples
N52E	P48 (NS1/2)	N/A	Yes
S53P	P48 (NS1/2)	N/A	No
K165R	P48 (NS1/2)	N/A	Yes
S644P	NTPase (NS3)	N/A	No
R731K	P22 (NS4)	N/A	Yes
K750R	P22 (NS4)	N/A	Yes
P845Q	P22 (NS4)	N/A	Yes
A853T	P22 (NS4)	N/A	Yes
V1057I	Protease (NS6)	N/A	Yes
D1362E	RdRp (NS7)	173	Yes
S1482T	RdRp (NS7)	293	Yes
V1521I	RdRp (NS7)	332	No
K1546Q	RdRp (NS7)	357	Yes
T1549A	RdRp (NS7)	360	Yes

Each nonsynonymous change that occurred along the branch in the ORF1 and RdRp phylogenetic trees leading to the GII.P16-GII.4 Sydney 2012/GII.3/GII.2 clade is shown. N/A—not applicable.

## Discussion

Here, we demonstrate that the emerging GII.P16-GII.4 Sydney 2012 norovirus lineage is circulating in the UK and USA, in addition to previous reports of circulation in Asia and Germany [6–8]. Analysis of available sequences suggests that this lineage has been circulating since October 2014 or earlier (Fig 2). The lack of amino acid substitutions in the capsid suggests that this lineage will not be able to escape existing herd immunity generated against Sydney 2012 since its emergence as a pandemic in 2012. However, previous studies have implicated the RdRp as an important component of viral fitness and demonstrated that RdRp changes can influence viral transmission by modulating the replication fidelity, and thus the viral diversity [14,15]. Several of the changes in the RdRp are in the palm subunit that contains most of the catalytic residues (Fig 3). Little is currently known about whether changes in the other proteins encoded by ORF1 have the ability to influence viral transmission. However, as the GII.P16 RdRp found with the GII.4 Sydney 2012 capsid has acquired several nonsynonymous changes close to positions known to influence RdRp function and viral transmission, this RdRp may have different properties and/or a greater ability to transmit compared with other GII.P16 RdRps. Only four of the RdRp changes occur in the genome region sequenced from the GII.P16-GII.2 virus that recently dominated norovirus outbreaks in Germany [8]. However, all four of these changes are also present in the GII.P16-GII.2 virus. We therefore suggest that the worldwide circulation of the GII.P16-GII.4 Sydney 2012 lineage and the high prevalence of the GII.P16-GII.2 virus are due to increased transmissibility driven by RdRp changes in GII.P16. Importantly, the GII.2 capsid is typically rare and is therefore unlikely to be as fit as the prevalent GII.4 capsid. It is therefore possible that combining an advantageous RdRp with the GII.4 Sydney 2012 capsid may result in a highly transmissible virus, despite the lack of antigenic changes in the capsid. Importantly, surveillance strategies based on capsid genotyping alone would be unable to distinguish between the GII.P16-GII.4 Sydney 2012 lineage and other Sydney 2012 viruses. It is therefore vital that surveillance efforts genotyping both the capsid and RdRp assess whether the GII.P16-GII.4 Sydney 2012 lineage replaces the GII.Pe-GII.4 Sydney 2012 strain over the coming months.

## Supporting information

S1 File Alignment of GII.P16 RdRp sequences. This alignment was used to reconstruct the GII.P16 phylogenetic tree in Fig 1.
(FASTA)

S2 File Alignment of GII.4 Sydney 2012 capsid sequences. This alignment was used to reconstruct the GII.4 Sydney 2012 phylogenetic trees in Fig 2.
(FASTA)

S3 File Phylogenetic tree of GII.P16 RdRp sequences. The BEAST maximum clade credibility tree used in Fig 1.
(NEX)

S4 File Phylogenetic tree of GII.4 Sydney 2012 sequences. The BEAST maximum clade credibility tree used in Fig 2.
(NEX)

S5 File BEAST XML file for GII.P16 analysis. (XML)

S6 File BEAST XML file for GII.4 Sydney 2012 analysis. (XML)
